# Supplementary material for: Molecular dynamics simulations of a multicellular model with cell-cell interactions and Hippo signaling pathway
Source: PLoS Comput Biol. 2024 Nov 11;20(11):e1012536. doi: 10.1371/journal.pcbi.1012536 (PMC11554158; doi:10.1371/journal.pcbi.1012536)
Supplement: S1 Appendix — (PDF) [file pcbi.1012536.s005.pdf]

S1 Appendix. Derivation and Simulation of the Computational Model

Toshihito UMEGAKI, Hisashi MORIIZUMI, Fumiko OGUSHI,
Mutsuhiro TAKEKAWA and Takashi SUZUKI

A1 Introduction

This appendix provides a detailed explanation of the computational model used to simulate the time evolution of nuclear YAP/TAZ concentrations and other computational results described in this paper. The model incorporates cell growth, division, and interactions between cells within a multicellular environment, combining physical mechanics with biochemical reaction kinetics. The simulation is conducted at regular time intervals, with the physical and chemical states of the cells updated at each step.

A2 Model Parameters

The model employs several parameters that control the physical and chemical processes in the simulation. The key parameters include:

1. **Total Time Steps and Output Time Steps**: These determine the number of divisions of time throughout the simulation.
2. **Initial Cell Mass**: The initial mass of the cells, m_0 is set to 2.0×10^{-12} kg, and this mass increases as the cells grow.
3. **Friction Coefficient**: This represents the resistance force to cell movement and k_{fric} is set to 5×10^{-13} kg/s.
4. **Energy Scale of the Lennard-Jones Potential**: This energy parameter, ϵ, set to 2×10^{-15} kgμm^2/s^2, determines the attractive and repulsive forces between cells.
5. **Time Step**: The simulation time interval is set to 3.75 seconds.
6. **Initial Cell Diameter**: The initial cell diameter, σ' is set to 5.0 μm, and it increases as the cells grow.
7. **Reaction Constants**: These parameters, a_1, a_2, a_3, and b_1 with values shown in Table A1 determine the rate of biochemical reactions that influence nuclear YAP/TAZ concentrations.

A3 Setting Initial Conditions

At the beginning of the simulation, the following initial conditions are set:

1. **Initial Cell Positions and Velocities**: Each cell is placed within the space using randomly generated spherical coordinates (angles and azimuths). These coordinates are converted to Cartesian coordinates and used in the simulation. The velocities are calculated based on the initial cell placements.
2. **Initial Cell States**: Each cell is initialized with an initial size, mass, and concentration of YAP/TAZ cytoplasm, YAP/TAZ nucleus, and P-YAP/TAZ cytoplasm (denoted as X_1, X_2, and X_3, respectively). These parameters change as the cells grow and divide.

Table A1: **Parameters for the reaction equations [1–3].**

Parameter	Definition	Value [unit]
Concentration	X_0: input transmitted into a cell when ρ_i=1	0.15 [μM]
Initial concentration	X^0_{1i}: YAP/TAZ cytoplasm<br>in normal tissues<br>in cancer tissues	 0.05 [μM]<br>0.1 [μM]
	X^0_{2i}: YAP/TAZ nucleus<br>in normal tissues<br>in cancer tissues	 0.2 [μM]<br>0.4 [μM]
	X^0_{3i}: P-YAP/TAZ cytoplasm	0 [μM]
Reaction rate	a_1: YAP/TAZ cytoplasm $\rightarrow$ nucleus	5 $\times 10^{-2}[/s]$
	a_2: YAP/TAZ nucleus $\rightarrow$ cytoplasm	5 $\times 10^{-4}$ [/s]
	b_1: YAP/TAZ cytoplasm<br>$\rightarrow$ P-YAP/TAZ	 1 [/(μM$\cdot$ s)]
	a_3: P-YAP/TAZ<br>$\rightarrow$ YAP/TAZ cytoplasm	 1 $\times 10^{-4}$ [/s]

A4 Time Evolution Algorithm

The simulation progresses through time using the following steps:

1. **Cell Growth and Division**:
   - As the simulation progresses, cells grow according to their nuclear YAP/TAZ concentration. If the concentration $X_{2,i}$ exceeds a certain threshold X_{th}, the cells continue to grow and eventually divide once σ_i^k reach a sufficient size $\sqrt[3]{2}\sigma_0$.
   - The daughter cells inherit the properties of the parent cells but are positioned in a direction determined randomly.
2. **Interactions Between Cells**:
   - The forces between cells are calculated using the Lennard-Jones potential, which models both attractive and repulsive forces based on the distance between cells.
   - The calculated forces are used to update the cells' velocities and positions using the Verlet integration method, which efficiently tracks the movement of cells at each time step.
3. **Biochemical Reaction Simulation**:
   - Changes in nuclear YAP/TAZ concentrations are updated based on reaction kinetics equations solved using the Runge-Kutta method. This method numerically solves complex reaction systems in the simulation.
   - The reaction rates depend on cell density, which is calculated from the distances between cells. This models the situation where reaction rates vary spatially.

A5 Output data

During the simulation, the following data is output at regular intervals:

- **Cell Positions and Sizes**: The positions and sizes of all cells are recorded at each time step, allowing tracking of cell movements and growth patterns.
- **Nuclear YAP/TAZ Concentrations**: The maximum, minimum, and average concentrations of nuclear YAP/TAZ, X_{2i} in i-th cell are recorded and analyzed over time.
- **The ratio of X_2 between cancer and normal cells**: The ratio of X_2 averaged across multiple cells between cancer and normal cells is tracked throughout the simulation.

A6 Numerical Algorithms and Subroutines

Several important numerical algorithms and subroutines are used in the simulation:

- **Eigenvalue Problem Solver**: An eigenvalue problem is numerically solved to compute the reaction kinetics of nuclear YAP/TAZ concentrations, allowing efficient computation of the system's evolution.
- **Matrix-Vector Multiplication and Matrix Inversion**: Matrix operations are frequently performed during the biochemical reaction simulation, essential for calculating interactions between cells and reaction rates.
- **Uniform Random Number Generation**: Uniform random numbers are generated for setting initial conditions and determining positions during cell division. This randomness introduces diversity in cell placement and behavior in the simulation.

A7 Conclusion

This appendix provides a detailed description of the derivation and implementation of the computational model used to simulate the time evolution of nuclear YAP/TAZ concentrations and other results in a multicellular environment. By combining physical mechanics and biochemical reaction kinetics, the model accurately captures the complex behavior of cell populations and offers new insights into the regulation of YAP/TAZ in different cellular contexts.

References

[1] B. Zhao, X. Wei, W. Li, R. Udan, Q. Yang, J. Kim, J. Xie, T. Ikenoue, J. Yu, L. Li, P. Zheng, K. Ye, A. Chinnaiyan, G. Halder, Z. Lai, and K. Guan, Inactivation of YAP oncoprotein by the Hippo pathway is involved in cell contact inhibition and tissue growth control, *Genes and Development* **21**, 2747–2761, 2007.

[2] L. Guo, Y. Chen, J. Luo, J. Zheng, and G. Shao, YAP1 overexpression is associated with poor prognosis of breast cancer patients and induces breast cancer tissues growth by inhibiting PTEN, *FEBS Open Bio* **9** 437–445, 2019.

[3] K. Aoki, M. Yamada, K. Kunida, S. Yasuda, M. Matsuda, Processive phosphorylation of ERK MAP kinase in mammalian cells, *Proc. Natl. Acad. Sci.* **108**, 31, 12675–12680, 2011.
