# Supplementary material for: Molecular dynamics simulations of a multicellular model with cell-cell interactions and Hippo signaling pathway
Source: PLoS Comput Biol. 2024 Nov 11;20(11):e1012536. doi: 10.1371/journal.pcbi.1012536 (PMC11554158; doi:10.1371/journal.pcbi.1012536)
Supplement: S2 Appendix — (PDF) [file pcbi.1012536.s006.pdf]

S2 Appendix. Method to solve the kinetic equations using Verlet method.

Toshihito UMEGAKI, Hisashi MORIIZUMI, Fumiko OGUSHI,
Mutsuhiro TAKEKAWA and Takashi SUZUKI

We calculated the dynamics of i-th cell of i=1, $\cdots$, N^k at time t=kdt and $k = 1, \cdots , L$ by numerically solving the following time differential equations [1]:

$$m_i \frac{d\boldsymbol{v}_i}{dt} = \boldsymbol{f}_i + \boldsymbol{g}_i, \tag{B1 a}$$

$$\frac{d\boldsymbol{r}_i}{dt} = \boldsymbol{v}_i, \tag{B1 b}$$

where m_i is the mass of the i-th cell proportional to the cell volume, m_i=m_0 $(\sigma'_i/\sigma'_0)^3$. In order to integrate these equations (B1), we employed Verlet method [2] providing an efficient and accurate way to simulate the system's behavior over time. σ'_i is the cell radius of the i-th cell, as expressed as

$$\sigma'_i = \zeta\sigma_i, \tag{B2 a}$$

$$\zeta = \sqrt[6]{2 - \alpha_{\mathrm{LJ}}(1-\lambda)^2}, \tag{B2 b}$$

grows over time in growing cells. and $\boldsymbol{f}_i$ and $\boldsymbol{g}_i \in \mathbb{R}^3$. $\boldsymbol{f}_i$ is the intercellular force acting on the ith cell as follows:

$$\boldsymbol{f}_i = \sum_{\substack{j=1 \\ j\neq i}}^{N} \sum_{\bar{r}_{ij}<r_{\mathrm{cut}}} \frac{-\mathrm{d}U(\bar{r}_{ij})}{\mathrm{d}\bar{r}_{ij}} \hat{\boldsymbol{r}}_{ij}, \tag{B3 a}$$

$$\bar{r}_{ij} = |\boldsymbol{r}_i - \boldsymbol{r}_j|, \tag{B3 b}$$

$$\hat{\boldsymbol{r}}_{ij} = \frac{\boldsymbol{r}_i - \boldsymbol{r}_j}{\bar{r}_{ij}}, \tag{B3 c}$$

$$r_{\mathrm{cut}} = 3\tilde{\sigma}_{ij}, \tag{B3 d}$$

$$\tilde{\sigma}_{ij} = \sigma_i + \sigma_j. \tag{B3 e}$$

T_{M} is the mitotic (M) phase and r_{cut} is the cutoff distance, where the potential force between i-and j-th cells is approximately 0. $\boldsymbol{g}_i$ is the friction force as follows:

$$\boldsymbol{g}_i = -k_{fric}\boldsymbol{v}_i. \tag{B4}$$

Previous studies have modeled one cell as approximately a sphere. In some cases, the force acting between cells is assumed to be an elastic force [3] or a potential gradient [4]. In this study, to represent cell adhesion and detachment, we assume a potential $U(\bar{r}_{ij})$ as the Lennard-Jones (12-6) potential with softcore character [1,5] as follows:

$$U(\bar{r}_{ij}) = \lambda 4\epsilon \left\{ \frac{1}{\left[\alpha_{\mathrm{LJ}}(1-\lambda)^2 + (\bar{r}_{ij}/\tilde{\sigma}_{ij})^6\right]^2} - \frac{1}{\alpha_{\mathrm{LJ}}(1-\lambda)^2 + (\bar{r}_{ij}/\tilde{\sigma}_{ij})^6} \right\}, \tag{B5 a}$$

$$\bar{r}_{ij} = |\boldsymbol{r}_i - \boldsymbol{r}_j|, \tag{B5 b}$$

$$\tilde{\sigma}_{ij} = \sigma_i + \sigma_j, \tag{B5 c}$$

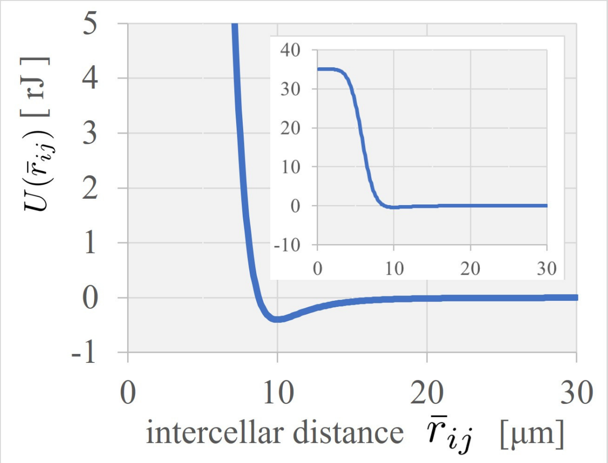

Figure B1: **Lennard-Jones (12-6) potential with a softcore character [5].** The softcore character represents an elastic wall so that the potential at a short range does not suddenly increase. This distribution is calculated using Eq. (B5 a) with parameters in Tab B1.

where $\tilde{\sigma}_{ij}$=σ_i+σ_j is proportional to the sum of the radii of i th and jth cells, and ϵ represents the binding energy (dissociation energy) between two cells. The softcore characteristic refers to an elastic wall, which prevents a sudden increase in potential forces due to the contiguity between cells. The potential of Eq. (B5 a) is characterized by the cell or collision radii. Sums of σ_i'+σ_j' and σ_i''+σ_j'' can be defined by the gradient of the potential $\mathrm{d}U(\bar{r}_{ij})/\mathrm{d}\bar{r}_{ij}$=0 and the potential $U(\bar{r}_{ij})$=0, respectively, where σ_i'' is the cell collision radius, expressed as follows:

$$\sigma_i'' = \sqrt[6]{1-\alpha_{\mathrm{LJ}}(1-\lambda)^2}\sigma_i. \tag{B6}$$

Figure B1 shows the distribution of Lennard-Jones (12-6) potential with a softcore character calculated

Table B1: **Parameters for MD equations.**

Parameter	Definition	Value [unit]
Single cell after division	m_0: mass	2 [ng] [6,7]
	$\sigma_0{}'$: cell radius ($= \sigma_0 \sqrt[6]{2-\alpha_{\mathrm{LJ}}(1-\lambda)^2}$)	5 [μm]
Cell cycle	T_{CC}: Whole period	20 [h]
	T_M: Mitotic period	1 [h] [8]
Frictional force	k_{fric}: Frictional coefficient	5×10^{-13} [kg/sec]
Potential of a cell	ϵ: Positive parameter	2 [rJ]
	λ: Coupling parameter	0.2 [5]
	α_{LJ}: Parameter	0.3 [5]
	$\lambda\epsilon$: Depth at the potential minimum	0.4 [rJ]
	$2\sigma_0$: Intercellular distance (potential=0)	9.06 [μm]
Cell growth	X_{th}: Threshold, cell growth if $X_{2i} > X_{\mathrm{th}}$	0.012 [μM]

using Eq. (B5 a) parameters in Table B1, where m_0 is mass and σ_0' is the cell radius of single cell after division [6, 7], T_{CC} and T_M are whole and mitotic periods of cell cycles set experimentally [8], k_{fric} is a frictional coefficient, $\lambda\epsilon$ is depth at the potential minimum, and $2\sigma_0$ is intercellular distance (potential = 0). determined from a previous study [5].

The single-cell mass after division, m_0, has been set to 2 ng by considering an intermediate value between 1 and 3-4 ng from references [6, 7]. The other parameters have been empirically adjusted for calculations. The unit [rJ] is the ronto joule (ronto=10^{-27}).

Algorithm B1 Solutions of the kinetic equations solved using Verlet method

Require: $\boldsymbol{r}_i, \boldsymbol{v}_i, i = 1, \cdots N$

Require: $\zeta = \sqrt[6]{2 - \alpha_{\mathrm{LJ}}(1-\lambda)^2}$,

1: $\bar{r}_{ij} = |\boldsymbol{r}_i - \boldsymbol{r}_j|$, i, j=1,$\cdots N$

2: $\tilde{\sigma}_{ij} = \sigma_i + \sigma_j$, i, j=1,$\cdots N$

3: $\sigma_i{}' = \sigma_i/\zeta$,

4: $U(\bar{r}_{ij}) = \lambda 4\epsilon \left\{ 1/\left[\alpha_{\mathrm{LJ}}(1-\lambda)^2 + (\bar{r}_{ij}/\tilde{\sigma}_{ij})^6\right]^2 - 1/\left[\alpha_{\mathrm{LJ}}(1-\lambda)^2 + (\bar{r}_{ij}/\tilde{\sigma}_{ij})^6\right] \right\}$,

5: $\boldsymbol{f}_i = \sum_{\substack{j=1 \\ j\neq i}}^{N} \sum_{\bar{r}_{ij}<r_{\mathrm{cut}}} -\mathrm{d}U(\bar{r}_{ij})/\mathrm{d}\bar{r}_{ij}\hat{\boldsymbol{r}}_{ij}, i = 1, \cdots, N$

6: $\boldsymbol{g}_i$=-k_{fric} $\boldsymbol{v}_i$, i=1,$\cdots N$

7: $\boldsymbol{v}_i \leftarrow \boldsymbol{v}_i + dt(\boldsymbol{f}_i + \boldsymbol{g}_i)/2m_i \cdots$ velocity update (1st of 2 steps),

8: $\boldsymbol{r}_i \leftarrow \boldsymbol{r}_i + dt\boldsymbol{v}_i \cdots$ position update,

9: $\boldsymbol{f}_i = \sum_{\substack{j=1 \\ j\neq i}}^{N} \sum_{\bar{r}_{ij}<r_{\mathrm{cut}}} -\mathrm{d}U(\bar{r}_{ij})/\mathrm{d}\bar{r}_{ij}\hat{\boldsymbol{r}}_{ij}, i = 1, \cdots, N$

10: $\boldsymbol{g}_i$ = -k_{fric} $\boldsymbol{v}_i$, i=1,$\cdots, N$

11: $\boldsymbol{v}_i \leftarrow \boldsymbol{v}_i + dt(\boldsymbol{f}_i + \boldsymbol{g}_i)/2m_i \cdots$, velocity update (2nd of 2 steps),

Ensure: $\boldsymbol{r}_i, \boldsymbol{v}_i, i = 1, \cdots, N$

References

[1] H. Gould and J. Tobochnik, An Introduction to Computer Simulation Methods: Applications to Physical Systems, *Addison-Wesley*, 1988.

[2] D. Frenkel and B. Smit, *Understanding Molecular Simulations*, Academic Press, San Diego, 1996.

[3] A. Malmi-Kakkada, X. Li, H. Samanta, S. Sinha, D. Thirumalai, Cell growth rate dictates the onset of Glass to Fluidlike transition and long-term Superdiffusion in an evolving cell colony, *Physical Review X* **8**, 021025, 2018.

[4] I. Ramis-Conde, M. Chaplain, A. Anderson, Mathematical modelling of cancer cell invasion of tissue, *Mathematical and Computer Modelling* **47**, 533–545, 2008.

[5] T. C. Beutler, A. E. Mark, R. C. Schaik, P. R. Gerber, W. F. Gunsteren, Avoiding singularities and numerical instabilities in free energy calculations based on molecular simulations, *Chemical Physics Letter* **222**, 529-539, 1994.

[6] E. Bianconi, A. Piovesan, F. Facchin, A. Beraudi, R. Casadei, F. Frabetti, L. Vitale, M. Pelleri, S. Tassani, F. Piva, S. Perez-Amodio, P. Strippoli and S. Canaider, An estimation of the number of cells in the human body, *Ann. Hum. Biol.*, **40**, 6, 463–471, 2013.

[7] C. Sims and N. Allbritton, Analysis of single mammalian cells on-chip, *Lab Chip*, **7**, 2007, 423–440.

[8] M. Mitsushima, K. Aoki, M. Ebisuya, S. Matsumura, T. Yamamoto, M. Matsuda, F. Toyoshima and E. Nishida, Revolving movement of a dynamic cluster of actin filaments during mitosis *Journal of Cell Biology* **191**, 3, 453–462, 2010.
