# Supplementary material for: Molecular dynamics simulations of a multicellular model with cell-cell interactions and Hippo signaling pathway
Source: PLoS Comput Biol. 2024 Nov 11;20(11):e1012536. doi: 10.1371/journal.pcbi.1012536 (PMC11554158; doi:10.1371/journal.pcbi.1012536)
Supplement: S3 Appendix — (PDF) [file pcbi.1012536.s007.pdf]

S3 Appendix. Method to solve reaction equations using eigenvalue problem.

Toshihito UMEGAKI, Hisashi MORIIZUMI, Fumiko OGUSHI, Mutsuhiro TAKEKAWA and Takashi SUZUKI

If we rewrite the reaction equations by focusing only on the i-th cell and omitting the subscript i, we obtain the following reaction equations and calculate the time evolutions of the concentrations X_1, X_2, and X_3 with the packing fraction ρ.

$$\frac{dX_1}{dt} = -(a_1 + b_1\rho X_0)X_1 + a_2X_2 + a_3X_3, \quad \text{(C1 a)}$$

$$\frac{dX_2}{dt} = +a_1X_1 - a_2X_2, \quad \text{(C1 b)}$$

$$\frac{dX_3}{dt} = +b_1\rho X_0X_1 - a_3X_3, \quad \text{(C1 c)}$$

The reaction equations Eq. (C1) can be summarized in vector $\boldsymbol{X}$ and matrix A as follows:

$$\frac{d\boldsymbol{X}}{dt} = \mathrm{A}\boldsymbol{X}, \quad \text{(C2 a)}$$

$$\boldsymbol{X} = \begin{pmatrix} X_1 \\ X_2 \\ X_3 \end{pmatrix}, \quad \text{(C2 b)}$$

$$\mathrm{A} = \begin{pmatrix} -a_1 - b_1\rho X_0 & a_2 & a_3 \\ a_1 & -a_2 & 0 \\ b_1\rho X_0 & 0 & -a_3 \end{pmatrix} \quad \text{(C2 c)}$$

If we define a matrix with the eigenvectors of A as column vectors P, then the matrix Λ with eigenvalues as diagonal components is formulated as follows:

$$\mathrm{A} = \mathrm{P}\Lambda\mathrm{P}^{-1} \quad \text{(C3 a)}$$

$$\Lambda = \begin{pmatrix} \lambda_1 & 0 & 0 \\ 0 & \lambda_2 & 0 \\ 0 & 0 & \lambda_3 \end{pmatrix} \quad \text{(C3 b)}$$

We define $\boldsymbol{Y}$ as $\mathrm{P}^{-1}\boldsymbol{X}$ and multiply P^{-1} from the left side of Eq. (C2 a) to obtain the following equation:

$$\frac{d\boldsymbol{Y}}{dt} = \Lambda\boldsymbol{Y}, \quad \text{(C4 a)}$$

$$\boldsymbol{Y} = \mathrm{P}^{-1}\boldsymbol{X}, \quad \text{(C4 b)}$$

$$\boldsymbol{X} = \mathrm{P}\boldsymbol{Y} \quad \text{(C4 c)}$$

Dividing the vector $\boldsymbol{Y}$ into scalar components Y_i gives the following equation.

$$\frac{dY_i}{dt} = \lambda_iY_i, \quad i = 1, 2, 3 \quad \text{(C5)}$$

The differential equation Eq. (C5) has the following solution:

$$Y_i = Y_i^0 \exp(\lambda_i t), \quad i = 1, 2, 3 \quad \text{(C6)}$$

Multiplying matrix P by the right-hand side of Eq. (C3 a) and defining the i-th column vector of matrix P as $\boldsymbol{p}_i$ and identity matrix as I, we obtain the following equation:

$$(\lambda_i \mathrm{I} - \mathrm{A})\, \boldsymbol{p}_i = \mathbf{0}, \quad i = 1, 2, 3 \tag{C7}$$

To find $\boldsymbol{p}_i$ such that $\boldsymbol{p}_i \neq \mathbf{0}$, the determinant on the left side of Eq. (C7) must be zero. That is, $det(\lambda_i \mathrm{I} - \mathrm{A})$=0:

$$\begin{vmatrix} \lambda_i + a_1 + b_1 \rho X_0 & -a_2 & -a_3 \\ -a_1 & \lambda_i + a_2 & 0 \\ -b_1 \rho X_0 & 0 & \lambda_i + a_3 \end{vmatrix} = 0, \tag{C8 a}$$

$$\leftrightarrow$$

$$\lambda_i \left[\lambda_i^2 + (a_1 + a_2 + a_3 + b_1 \rho X_0)\, \lambda_i + (a_1 + a_2) a_3 + a_2 b_1 \rho X_0 \right] = 0 \tag{C8 b}$$

The eigenvalues λ_1 and λ_2 were obtained from the solution of the quadratic equation, Eq. (C8), and λ_3=0.

$$\lambda_1 = \frac{-(a_1 + a_2 + a_3 + b_1 \rho X_0) + \sqrt{(a_1 + a_2 + a_3 + b_1 \rho X_0)^2 - 4\left[(a_1 + a_2) a_3 + a_2 b_1 \rho X_0\right]}}{2}, \tag{C9 a}$$

$$\lambda_2 = \frac{-(a_1 + a_2 + a_3 + b_1 \rho X_0) - \sqrt{(a_1 + a_2 + a_3 + b_1 \rho X_0)^2 - 4\left[(a_1 + a_2) a_3 + a_2 b_1 \rho X_0\right]}}{2}, \tag{C9 b}$$

$$\lambda_3 = 0 \tag{C9 c}$$

Writing the second and third lines of equation Eq. (C7), we obtain the following equations:

$$-a_1 p_{1,i} + (\lambda_i + a_2) p_{2,i} = 0 \tag{C10 a}$$

$$-b_1 \rho X_0 p_{1,i} + (\lambda_i + a_3) p_{3,i} = 0 \tag{C10 b}$$

We obtain the eigenvector $\boldsymbol{p}_i$ from (C10).

$$\boldsymbol{p}_i = \begin{pmatrix} p_{1,i} \\ p_{2,i} \\ p_{3,i} \end{pmatrix} = \begin{pmatrix} (\lambda_i + a_3) \\ a_1(\lambda_i + a_3)/(\lambda_i + a_2) \\ b_1 \rho X_0 \end{pmatrix} \tag{C11}$$

Matrix P can summarize the column vectors $\boldsymbol{p}_i$ of Eq. (C11), as follows:

$$\mathrm{P} = \begin{pmatrix} (\lambda_1 + a_3) & (\lambda_2 + a_3) & (\lambda_3 + a_3) \\ a_1(\lambda_1 + a_3)/(\lambda_1 + a_2) & a_1(\lambda_2 + a_3)/(\lambda_2 + a_2) & a_1(\lambda_3 + a_3)/(\lambda_3 + a_2) \\ b_1 \rho X_0 & b_1 \rho X_0 & b_1 \rho X_0 \end{pmatrix} \tag{C12}$$

From the above equation, $\boldsymbol{X}(t)$ can be obtained as follows:

$$\boldsymbol{X}(t) = \mathrm{P} \boldsymbol{Y}(t) \tag{C13 a}$$

$$\boldsymbol{Y}(t) =^t \left[{}^t\boldsymbol{Y}^0 \mathrm{E(t)} \right] \tag{C13 b}$$

$$\mathrm{E(t)} = \begin{pmatrix} \exp(\lambda_1 t) & 0 & 0 \\ 0 & \exp(\lambda_2 t) & 0 \\ 0 & 0 & \exp(\lambda_3 t) \end{pmatrix} \tag{C13 c}$$

$$\boldsymbol{Y}^0 = \mathrm{P}^{-1} \boldsymbol{X}^0 \tag{C13 d}$$

Figure C1 shows ρ-dependencies of the eigenvalues λ_1 and λ_2 calculated using EVP. When ρ=0-1.5, λ_1 and λ_2 are -0.0001 $\sim$ -0.0004 [/s] and -0.1 $\sim$ -0.2 [/s], respectively. In this calculation example, λ_1 and λ_2 are real numbers, but if the inside terms in the square roots are negative in Eq (C9 a) and Eq (C9 b), in eigenvalues, λ_1 and λ_2 become complex numbers, and the concentration oscillates periodically according to solutions Y_i. Therefore, we mathematically explore methods for controlling this phenomenon through drug therapy, such as adjusting the timing of inhibitor administration to match the oscillation periods.

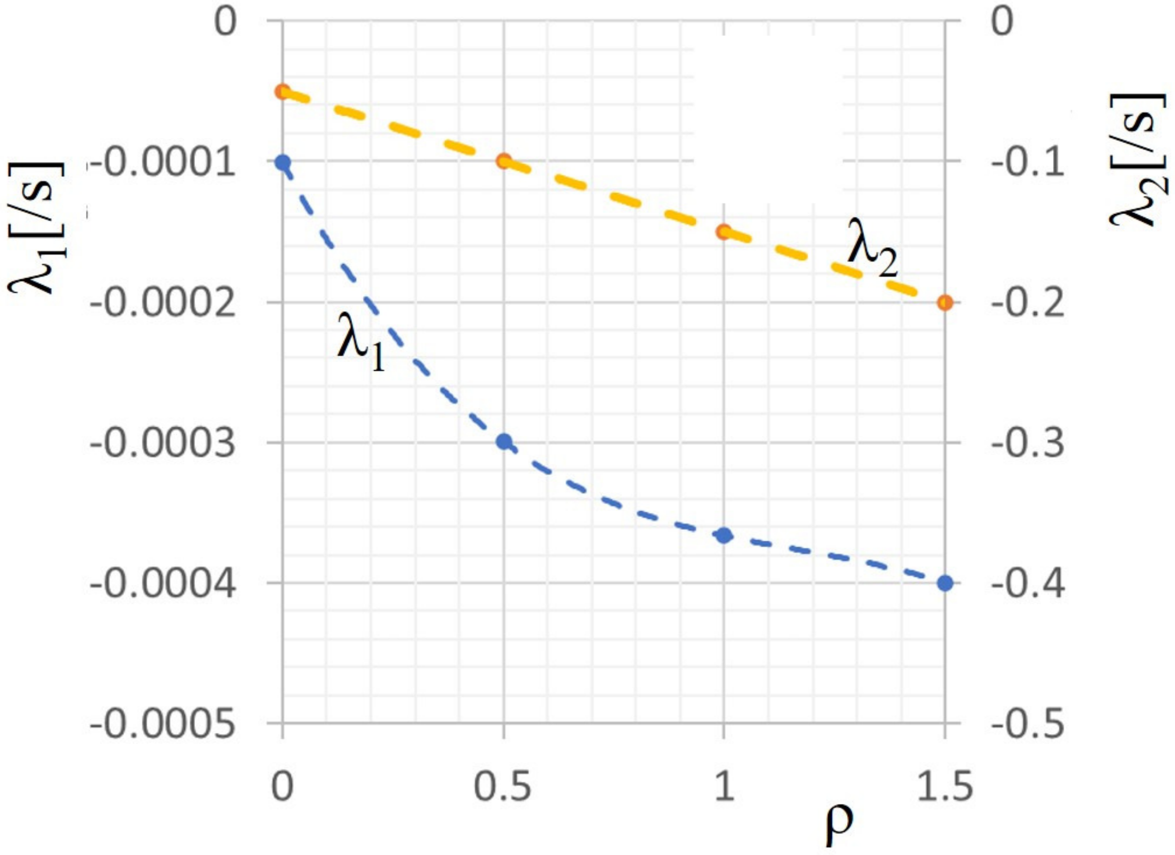

Figure C1: **Packing fraction dependencies of eigenvalues λ_1 and λ_2.**
