# Supplementary material for: Molecular dynamics simulations of a multicellular model with cell-cell interactions and Hippo signaling pathway
Source: PLoS Comput Biol. 2024 Nov 11;20(11):e1012536. doi: 10.1371/journal.pcbi.1012536 (PMC11554158; doi:10.1371/journal.pcbi.1012536)
Supplement: S4 Appendix — (PDF) [file pcbi.1012536.s008.pdf]

S4 Appendix. Packing fraction of each cells.

Toshihito UMEGAKI, Hisashi MORIIZUMI, Fumiko OGUSHI,
Mutsuhiro TAKEKAWA and Takashi SUZUKI

As an index representing cell density in multicellular systems, we define the packing fraction of i-th cell, ρ_i, as follows:

$$\rho_i = \beta \sum_{\substack{j=1 \\ j \neq i}}^{N} \exp\left(-\bar{r}_{ij}/\sigma_0'\right), \tag{D1 a}$$

$$\bar{r}_{ij} = \left|\boldsymbol{r}_i - \boldsymbol{r}_j\right|, i, j = 1, \cdots, N \tag{D1 b}$$

where ρ_i is assumed to be a sigmoid function with respect to α_i and ρ_i^{D} is the packing fraction of a daughter cell after division; σ_0' is the radius of the newly divided cell. $\bar{r}_{ij}$ is the intercellular distance between i and jth cells; N is the total number of cells in the tissue, and we set $\beta = \exp(2)/12$ such that ρ_i becomes 1 when the cells are surrounded by other cells, as shown in Fig. D1, which shows a multicellular arrangement of perspective (left) and cross-sectional (right) views when ρ_i=1, at which 12 cells (green) surround one cell (orange). The cell positions $\boldsymbol{r}_i$ can be calculated with MD calculations.

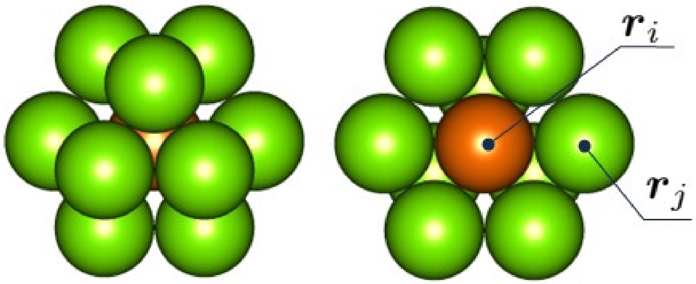

Figure D1: **Perspective (left) and cross-sectional (right) views of a cell layout of the multicell when ρ_i=1, at which 12 cells (green) surround one cell (orange).** $\boldsymbol{r}_i$ and $\boldsymbol{r}_j$ are center positions of the orange and green cells, respectively.
