# Supplementary material for: Molecular dynamics simulations of a multicellular model with cell-cell interactions and Hippo signaling pathway
Source: PLoS Comput Biol. 2024 Nov 11;20(11):e1012536. doi: 10.1371/journal.pcbi.1012536 (PMC11554158; doi:10.1371/journal.pcbi.1012536)
Supplement: Attachment — Submitted filename: PCOMPBIOL-D-23-02102.pdf [file pcbi.1012536.s009.pdf]

In this paper by Umegaki et al, the authors present their work describing their approach to employ a multiscale modeling framework to model cell-cell interactions originating in Hippo signaling networks.The authors describe a method of constructing a 3D model of cell proliferation in the YAP/TAZ Hippo pathway. An innovative aspect of this work is their proposed approach to concurrently solve the multicellular kinetic equations using molecular dynamics (MD) followed by the reaction equations in each cell of the multicellular system, and subsequently evaluating the cell-cell interactions. This is similar in spirit to similar recent work by A.N.F. Versypt (Curr. Opin 2019), SP Beik (Plos Comp Bio 2023), and work from the Rangamani lab at UCSD (among many others), which explore cell populations, making the topic timely and relevant for the journal. However, in its present form the paper has some significant flaws which decrease enthusiasm about this work. These issues are described below.

Major Issues:

1. The first main issue with this work has to do with the way in which it is presented. The introduction makes it sound like there will be a significant biological undertone to the paper, promising to provide some insight to explain why cells with high YAP/TAZ do not exhibit cell cycle arrest and continue to grow. However, the contributions of the work towards these insights are lacking. The results are not well explained and it is unclear whether the results prove or disprove the hypothesis.

2. It seems that the paper is strongly focused on the methodology and theoretical developments – appropriate for the journal, but the key mathematical and theoretical contributions are also not described clearly or placed in the context of other work. For example, it is unclear how this biochemical interaction model of complexes in 3D out-performs other existing models. What did we learn from this approach that we could not learn from a traditional ODE model? It is also unclear how the authors measure the performance of this 3D method compared to other models.

3. In the paper, the main focus is devoted to the methodology. If this is the case, then the relevance of the method and the importance in the context of other work should be presented.

4. The main results presented by the authors is that 2x the concentration of YAP/TAZ in cancer cells – relative to normal cells – is sufficient for cell proliferation to proceed unabated even when the $X_{th}>X_{2i}$. However, no mechanistic insights are provided or discussed about the potential impact for biology.

5. The MD component of the work seems novel and potentially relevant! How do the different scales "talk" to each other? The details are not presented in enough detail to assess their impact on the work.

6. The main finding from the paper abstract states: *"The results show that a twofold increase in YAP/TAZ levels of cancer cells was sufficient to evade cell cycle arrest compared to normal cells, enabling cells to continue proliferating even under high-density condition."*

Could the authors elaborate on this point? How is this finding significant? Was this already not known? Why was this novel approach necessary to capture these findings? Or is the exercise in this work about developing the method and the results confirm a known biological finding? More detail about the results would greatly strengthen the paper.

7. *"During the aging of the ith cell…"* indicates that the cells being modeled divide according to the volume, and not according to the timing, i.e. they divide when a certain size has been achieved, rather than when a certain amount of time has elapsed from the previous division point. Is that an accurate assumption for the cells being modeled here?

8. In the definition of dσ/dt, how is T_CC determined if the entire cell cycle hasn't progressed yet? It would be helpful to readers if the authors describe their assumptions for the model and their reasoning behind these assumptions.

9. The authors write: *"Instead, exponential cell proliferation continued for up to 200 h"*

- How would one expect this number to change given the initial YAP/TAZ concentration was two-fold greater?
- Should the simulation not have run for more than twice that amount of time? If not, what is the rationale for choosing that time-point to conclude that this is indicative of cancer dynamics? Isn't this just a linear effect?

  "Thus, a two-fold increase in YAP/TAZ levels was sufficient to override the density-dependent growth inhibition effect…"

What happens at the intermediate levels of YAP/TAZ concentrations? Is there a switch-like behavior where the malignancy appears, or is it a gradual increase with increasing YAP/TAZ levels?

Minor issues:
1. It was very difficult to follow the variables described in the paper. In general, the symbols are not well defined or explained throughout.
For instance,

- L is not defined anywhere before it is used to mark the index "k".
- γ is defined to be a value without an explanation about how it is obtained, or its physical significance.

〖- α〗 _i is not defined before its usage as the length of time of cell i that has elapsed.

- In section 2.6, the symbol ζ, which has not been defined before the usage, or immediately after. What is ζ?
- In Section 2.3, the quantity c_i is undefined?
- In Appendix 1, the term α_{LJ} is undefined, as is λ.

2. *"In the future, extending the mechanism of 3D dynamics from classical mechanics to quantum electrodynamics can…"*

This comment seems interesting but could cause confusion to readers. In general, the scale of a study should be appropriate to answer the biological questions at the necessary scale. If quantum electrodynamics are indeed needed, and this method could be leveraged for that goal, then it would help if the authors present some examples of this potential expansion of their work.

3. **Abstract:** *"We assume such 3D model with cell-cell interactions by simultaneously solving reaction and molecular dynamics (MD) equations by applying adhesion and repulsive forces that act between cells and frictional forces acting on each cell."*

This sentence is confusing. I think the authors state that their method simultaneously solves reaction dynamics and molecular dynamics to inform the cell-cell interactions. Other similar sentences are scattered throughout the paper.

4. **Materials and Methods:**
"Discrimine" should be "Discriminate"

Typo for “method”

Would be useful to have a one-line descriptor of the Verlet method here, as well as a citation.

5. **Section 2.2:** The phrase “rearrange their multicellularity” is unclear.

6. **Section 2.5:** Why is the packing fraction dependent on the position of the cell?

7. **Appendix 1:**
It would be helpful to explain the flow of the Verlet method for the readers to have a simple explanation of equation (A6), and how it is derived. While it is not necessary to provide details for the derivation, it would be helpful to at least walk the readers through the rationale for this expression.
〖- α〗 _LJ is only described as a “parameter” in Table A1. What is its physical significance?
- Similarly, ϵ is a parameter that is not defined.

Summary:
This work has significant of potential to present a novel methodology to combine simulations across multiple scales for biological applications but unfortunately the paper neither articulates the biological problem the authors are addressing clearly nor does it provide enough context for the novelty of the method. The mathematical and modeling work seems to be of great quality but the presentation needs to be improved.
