# Supplementary material for: Molecular dynamics simulations of a multicellular model with cell-cell interactions and Hippo signaling pathway
Source: PLoS Comput Biol. 2024 Nov 11;20(11):e1012536. doi: 10.1371/journal.pcbi.1012536 (PMC11554158; doi:10.1371/journal.pcbi.1012536)
Supplement: Attachment — Submitted filename: HippoPathwayMulticellsMDen240604.docx [file pcbi.1012536.s010.docx]

**Point-by-point response to referees’ issues**

First, we deeply thank all the reviewers for their positive and insightful comments and suggestions on the manuscript. In this point-by-point response letter, where deemed necessary, we have included our responses to the Decision from PLOS Computational Biology received on April 10, 2024, along with the revisions made to the manuscript. The manuscript has been revised with red and orange texts for Major and Minor Issues, respectively.

**Major Issues:**

**1. The first main issue with this work has to do with the way in which it is presented.**

**The introduction makes it sound like there will be a significant biological undertone to the paper, promising to provide some insight to explain why cells with high YAP/TAZ do not exhibit cell cycle arrest and continue to grow. However, the contributions of the work towards these insights are lacking. The results are not well explained and it is unclear whether the results prove or disprove the hypothesis.**

**Response:**

Thank you for your valuable comments regarding the presentation. To address Major Issue 1, we have incorporated the following texts, including a spatially restricted response to growth inhibition signals in cancerous conditions and the pivotal role of nuclear YAP/TAZ in regulating cellular proliferation dynamics, into each section within the brackets [] below:

[4 Discussion] “From Fig. 5, despite only a twofold increase in YAP/TAZ levels, the following observations can be made computationally and biologically:

(1) Tissue size increase: The data indicates an increase in overall tissue size, suggesting a dose-dependent effect of YAP/TAZ on tissue growth.

(2) Cell cycle arrest in normal vs. cancer cells: As seen in the upper section of Fig. 5, in normal cells, the cell cycle is arrested even in the peripheral regions of the tissue. In contrast, cancer cells in the same peripheral regions continue to proliferate, demonstrating a dysregulation in the normal cell cycle control mechanisms.

(3) Induction of cell cycle arrest in tissue cross-sections: The lower cross-section of Fig. 5 reveals that in normal cells, cell cycle arrest is induced as cells transition from the outer to the inner layers. However, in cancer cells, cell cycle arrest is predominantly induced in the central parts of the tissue only, highlighting a spatially restricted response to growth inhibition signals in cancerous conditions.

Moreover, Fig. 6 quantitatively demonstrates that cells typically double with each division, leading to exponential growth if unchecked. In normal tissues, this exponential increase is significantly suppressed. Remarkably, cancer cells with merely a twofold increase in YAP/TAZ levels exhibit almost no growth inhibition and continue to increase exponentially. This suggests that cancer cells with elevated YAP/TAZ levels bypass typical proliferative checks, underscoring the pivotal role of YAP/TAZ in regulating cellular proliferation dynamics in oncogenic contexts.”

[4 Discussion] “The ratio of X2 between cancer and normal cells (C/N X2mean) exhibits a temporal evolution in Fig. 8. Here, by setting the initial ratio of X2(cancer)/X2(normal) to 2, this ratio remains around 2 for up to 100 hours. However, after 100 hours, regions where the ratio approaches threefold emerge. This nonlinear region mediated by multicellularity, concerning YAP/TAZ concentration, can be seen as the reason why cancer cells continue to proliferate without showing cell cycle arrest.”

**2. It seems that the paper is strongly focused on the methodology and theoretical developments – appropriate for the journal, but the key mathematical and theoretical contributions are also not described clearly or placed in the context of other work. For example, it is unclear how this biochemical interaction model of complexes in 3D out-performs other existing models. What did we learn from this approach that we could not learn from a traditional ODE model? It is also unclear how the authors measure the performance of this 3D method compared to other models.**

**Response:**

Thank you for your comments regarding the theoretical development. In conventional models, cell density was not included as an input to the ODE model, but the current model focuses on the Hippo pathway and develops an ODE model with cell density as an input. This model enables the simulation of the relationship between cell density and proliferation, connected through the Hippo pathway.

Additionally, to emphasize novelty, we incorporated the following sentences into each section within the brackets [] below:

[Abstract] “Herein, a significant innovation in our approach is the introduction of a novel modeling component that includes cell division dynamics within the framework of the Hippo pathway’s influence on cell proliferation.”

[1 Introduction] “By integrating the molecular signaling of the Hippo pathway with cell mechanics and cell cycle dynamics, our study offers new insights into the mechanisms driving cancer proliferation and presents a more detailed and dynamic model compared to previous research.”

[2 Material and methods] “Discriminate cell division or nondivision, incorporating a novel model of the cell division influenced by dynamic intracellular signaling”

[2 Material and methods] “Our novel approach within the model includes a sophisticated algorithm for cell division based on real-time changes in the intracellular signaling pathways, particularly the Hippo-YAP/TAZ pathway.”

**3. In the paper, the main focus is devoted to the methodology. If this is the case, then the relevance of the method and the importance in the context of other work should be presented.**

**Response:**

We also would like to clarify relationships between the methodology and the references. We incorporated the following sentences into each section within “1 Introduction” below:

“However, these studies often did not directly address the intricate molecular signaling pathways that regulate cell proliferation and their interactions with mechanical forces at the multicellular level. Additionally, previous models did not incorporate the dynamic feedback mechanisms of the Hippo pathway on cell proliferation and survival, especially under high cell density conditions.”

“Our approach uniquely integrates the Hippo pathway signaling dynamics with cell-cell interactions, providing a more comprehensive understanding of the regulation of cell proliferation and tumorigenesis.”

“By integrating the molecular signaling of the Hippo pathway with cell mechanics and cell cycle dynamics, our study offers new insights into the mechanisms driving cancer proliferation and presents a newly and more dynamic model compared to previous research.” Here, 'mechanical force' refers to the force acting between cells to maintain a distance equivalent to the cell radius at equilibrium position. This force is expressed as a function of the intercellular distance, where an attractive force acts when the distance between cells is farther than the sum of the cell radii of two cells, and a repulsive force acts when the distance is nearer than that sum.

**4. The main results presented by the authors is that 2x the concentration of YAP/TAZ in cancer cells – relative to normal cells – is sufficient for cell proliferation to proceed unabated even when the X_th>X_2i. However, no mechanistic insights are provided or discussed about the potential impact for biology.**

**Response:**

Thank you for your comment towards a deeper insight into the mechanism. We have incorporated insights into the mechanisms of the following sentences into each section within the brackets [] below:

[Discussion] “The ratio of X2 between cancer and normal cells (C/N X2mean) exhibits a temporal evolution in Fig. 7. Here, by setting the initial ratio of X2(cancer)/X2(normal) to 2, this ratio remains around 2 when the time is up to 100 hours. Interestingly, when the time exceeds 100 hours, regions where the ratio approaches threefold emerge. This nonlinear region mediated by multicellularity, concerning nuclear YAP/TAZ concentration, can be seen as the reason why cancer cells continue to proliferate without showing cell cycle arrest.”

[Conclusion] ” Herein, a significant innovation in our approach is the introduction of a novel modeling component that inputs cell density, which reflects cell dynamics, into the Hippo pathway and enables the simulation of cell proliferation as the output response. This integration allows for a more detailed and dynamic representation of how cell division decisions are modulated by intracellular signaling.”

**5. The MD component of the work seems novel and potentially relevant! How do the**

**different scales “talk” to each other? The details are not presented in enough detail to**

**assess their impact on the work.**

**Response:**

Thank you for your questions regarding the details of MD. We would like to detail the "talk" with the MD reaction pathway. In MD calculations, the position *r*_i_ of the *i*-th cell is calculated, and using Eq (A20a), the cell-density ρ_i_ is determined. This ρ_i_ is then used to solve the Eqs (3a)-(3c) to obtain X_1i_ to X_3i_. Whether X_2i_ exceeds the threshold X_th_ determines whether the cell will proliferate or cease proliferation, thereby determining the size of the cell. If this size exceeds the cube root of 2, division occurs. The divided cells are repositioned, and the position *r*_i_ and velocity *v*_i_ of the cells are determined by MD. This feedback loop embodies the "talk" with the MD reaction pathway.

**6. The main finding from the paper abstract states: “The results show that a twofold increase in YAP/TAZ levels of cancer cells was sufficient to evade cell cycle arrest compared to normal cells, enabling cells to continue proliferating even under high-density condition.” Could the authors elaborate on this point? How is this finding significant? Was this already not known? Why was this novel approach necessary to capture these findings? Or is the exercise in this work about developing the method and the results confirm a known biological finding? More detail about the results would greatly strengthen the paper.**

**Response:**

Thank you for recommending the clarification of the main findings. The mathematical model used in this study revealed that a twofold increase in the YAP/TAZ levels of cancer cells allows them to evade cell cycle arrest and continue proliferating even under high-density conditions. Specifically, despite only a twofold increase in YAP/TAZ levels in cancer cells compared to normal cells, temporal observations of the nuclear YAP/TAZ levels, where YAP/TAZ functions, showed that the nuclear YAP/TAZ levels in cancer cells increased up to 2.86 times compared to normal cells. Previous studies have observed increased YAP/TAZ levels in cancer cells, but the nuclear levels, where YAP/TAZ functions, have not been sufficiently investigated. Our mathematical model demonstrated that as the overall YAP/TAZ levels in cells increase, the nuclear YAP/TAZ levels may increase even more. This study predicted the nuclear YAP/TAZ levels from the overall YAP/TAZ levels in cells. Interestingly, it was suggested that the nuclear YAP/TAZ levels are significantly affected by local density changes during cell division. YAP/TAZ is known to be involved in drug resistance, and by improving the accuracy of the mathematical model used in this study, it may be possible to predict the timing of nuclear YAP/TAZ level reduction in vivo and administer chemotherapy at that time to efficiently eliminate cancer cells. This approach could provide a new strategy to maximize the efficacy of chemotherapy while minimizing side effects.

**7. “During the aging of the ith cell…” indicates that the cells being modeled divide according to the volume, and not according to the timing, i.e. they divide when a certain size has been achieved, rather than when a certain amount of time has elapsed from the previous division point. Is that an accurate assumption for the cells being modeled here?**

**Response:**

Thank you for your question regarding the actual process of cell division. There are several prior studies that determine cell division based on cell size. For example, Zatulovskiy et al., have shown that cell growth during the G1 phase of the cell division cycle dilutes the cell cycle inhibitor Retinoblastoma protein (Rb) to trigger division in human cells in Science (2020). This study investigates how cell size influences the initiation of division from the G1 phase. On the other hand, Alberghina et al. have examined Saccharomyces cerevisiae, focusing on two thresholds: the critical cell size regulated by the carbon source required for entering cell division and the protein content per cell at the start of DNA replication (Ps) in Journal of Cell Biology (2004). It is found that cell division and DNA replication are determined by cell volume and protein amount, respectively. Additionally, we have added these two previous studies to the manuscript and included explanations that sequential cell division occurs when the cell size reaches the cube root of 2 times the size immediately after cell division.

**8. In the definition of dσ/dt, how is T_CC determined if the entire cell cycle hasn't**

**progressed yet? It would be helpful to readers if the authors describe their assumptions**

**for the model and their reasoning behind these assumptions.**

**Response:**

Thank you for your valuable comments regarding the model assumptions. We have defined cell cycle arrest with α_i_=constant → α_i_ aging at cell growth, α_i_ divides at *T*_CC_; *T*_CC_ = 20h: If in the cell-growth state of red or orange-colored cell for 20h, one cycle is defined, and added the text “With cell cycle arrest, α_i_ remains constant, while in cell growth, the age of the *i*-th cell, α_i_, increases. When α_i_ reaches time *T*_CC_, the cell divides. If the cell remains in a red or orange state over time *T*_CC_, it’s defined as one cycle.”

**9. The authors write: “Instead, exponential cell proliferation continued for up to 200 h”**

**- How would one expect this number to change given the initial YAP/TAZ concentration was two-fold greater?**

**- Should the simulation not have run for more than twice that amount of time? If not, what is the rationale for choosing that time-point to conclude that this is indicative of cancer dynamics? Isn't this just a linear effect?**

**“Thus, a two-fold increase in YAP/TAZ levels was sufficient to override the density-dependent growth inhibition effect…”**

**What happens at the intermediate levels of YAP/TAZ concentrations? Is there a switch-like behavior where the malignancy appears, or is it a gradual increase with increasing YAP/TAZ levels?**

**Response:**

Thank you for your comments regarding the simulation time. We attempted to double the current simulation time to investigate if it increased exponentially. Currently, the calculation time is 208 hours, during which periods, the total number of cells would reach 2^11 = 2048 after 11 divisions with assuming no cell proliferation arrest. On the other hand, with a doubled calculation time of 416 hours, the total number of cells would be 2^21=20,897,152, which is exponentially larger, causing an overflow and making the calculation impossible. Therefore, we extended the calculation time to 230 hours, 1.1 times the current time, and the calculation was successful.

Moreover, within this 230-hour calculation, it was observed that normal cells do not exhibit exponential cell proliferation beyond approximately 100 hours. This indicates that a calculation time twice as long is sufficient to investigate the possibility of exponential growth. Consequently, we have added the results of the calculation up to 230 hours to Discussion section as a semi-logarithmic plot of the temporal evolution of *N*(t). We have added red and blue solid lines, and orange dashed line in Fig. 7 in order to show time evolutions of the total number of multi-cells of cancer and normal tissues with (X^0^_1_, X^0^_2_)=(0.1, 0.4), (0.05, 0.2), respectively, and an exponential function with a time rounding-up parameter: exp ⌈t/20h⌉. From this semi-log plots, it is quantitatively confirmed that the red solid line for cancer cells almost matches with the exponential function of the orange dashed line up to about 200 hours, indicating that exponential cell growth continues up to about 200 hours. The basis for choosing this time point to draw conclusions is the nature of cancer cells, specifically as a model demonstrating exponential cell proliferation.

**Minor issues:**

**1. It was very difficult to follow the variables described in the paper. In general, the**

**symbols are not well defined or explained throughout.**

**For instance,**

**- L is not defined anywhere before it is used to mark the index “k”**

**- γ is defined to be a value without an explanation about how it is obtained, or its**

**physical significance.**

**〖- α〗 _i is not defined before its usage as the length of time of cell i that has**

**elapsed.**

**- In section 2.6, the symbol ζ, which has not been defined before the usage, or**

**immediately after. What is ζ?**

**- In Section 2.3, the quantity c_i is undefined?**

**- In Appendix 1, the term α_LJ is undefined, as is λ.**

**Response:**

Thank you for your helpful comments regarding the lack of explanation for variables. We have added the definitions for each parameter symbol 𝐿, γ, αi, ζ, ci, αLJ, and λ in the following text:

“L is the maximum number for k, and the time t is given by 𝑡=kΔt.”

“γ represents the dimensional ratio between the mother cell just before division and the daughter cells just after division, calculated by subtracting one from the cube root of two, such that the volume of one mother cell is equal to the sum of the volumes of the two daughter cells; =(2^1/3^−1)”

“where αi is the age of the i-th cell, that is, the time immediately after division, and cell colors of …”

“ci is the color index of the i-th cell, which is assigned the colors cyan, orange, blue, and red as 0, 1, 2, and 3, respectively.”

“With cell cycle arrest, αi remains constant, while in cell growth, the age of the i-th cell, αi , increases. When αi reaches time TCC, the cell divides. If the cell remains in a red or orange state over time TCC, it’s defined as one cycle.” “where ζ is the ratio of cell radius to the σi of the i-th cell; 𝜁 =[2−𝛼𝐿𝐽(1−λ)^2^]^1/6^, where αLJ is a parameter of the LJ potential, and λ is a coupling parameter.”

**2. “In the future, extending the mechanism of 3D dynamics from classical mechanics to**

**quantum electrodynamics can…”**

**This comment seems interesting but could cause confusion to readers. In general, the**

**scale of a study should be appropriate to answer the biological questions at the**

**necessary scale. If quantum electrodynamics are indeed needed, and this method**

**could be leveraged for that goal, then it would help if the authors present some**

**examples of this potential expansion of their work.**

**Response:**

Thank you for your advanced comments regarding the description of quantum electrodynamics. We plan to consider the effects of quantum electrodynamics in future research. However, since quantum electrodynamics was not actually applied in this paper, we have removed the text including quantum electrodynamics, "In the future … model," to avoid confusing the readers.

**3. Abstract: “We assume such 3D model with cell-cell interactions by simultaneously**

**solving reaction and molecular dynamics (MD) equations by applying adhesion and**

**repulsive forces that act between cells and frictional forces acting on each cell.”**

**This sentence is confusing. I think the authors state that their method simultaneously**

**solves reaction dynamics and molecular dynamics to inform the cell-cell interactions.**

**Other similar sentences are scattered throughout the paper.**

**Response:**

Thank you for your comments regarding Abstract and Author summary. To avoid confusion, "simultaneously" has been deleted from Abstract and Author summary.

**4. Materials and Methods:**

**“Discrimine” should be “Discriminate”**

**Typo for “method”**

**Would be useful to have a one-line descriptor of the Verlet method here, as well as a citation.**

**Response:**

“Discrimine” has been corrected to “Discriminate”, and a one-line descriptor of the Verlet method “which is a numerical technique used in molecular dynamics to integrate Newton's equations of motion, known for its accuracy and stability in long-term simulations. “ has been inserted with a citation [35] Frenkel1996AcademicPress.

**5. Section 2.2: The phrase “rearrange their multicellularity” is unclear.**

**Response:**

To clarify the expression "rearrange their multicellularity," it has been revised to "the multicellular structures are rearranged due to the equilibrium of forces acting among the cells."

**6. Section 2.5: Why is the packing fraction dependent on the position of the cell?**

**Response:**

To clarify the meaning of packing fraction, the phrase "which is defined as the sum of exponential functions of the distance between the i-th cell and other multicellular distances formulated in Appendix 3," has been inserted after "packing fraction ρi of the i-th cell."

**7. Appendix 1:**

**It would be helpful to explain the flow of the Verlet method for the readers to have a**

**simple explanation of equation (A6), and how it is derived. While it is not necessary to**

**provide details for the derivation, it would be helpful to at least walk the readers**

**through the rationale for this expression.**

**〖- α〗 _LJ is only described as a “parameter” in Table A1. What is its physical**

**significance?**

**- Similarly, ϵ is a parameter that is not defined.**

**Response:**

Thank you for your constructive comments regarding Appendix 1. To address Minor Issue 7, we have incorporated the following texts into Appendix 1 below:

"In order to integrate these equations (A1), we employed the Verlet method [35], providing an efficient and accurate way to simulate the system’s behavior over time" has been inserted after Eqs. (A1a) and (A1b). In order to integrate Eqs (A1a) and (A1b), we employed Verlet method [35] providing an efficient and accurate way to simulate the system’s behavior over time. ” ε represents the binding energy (dissociation energy) between two cells.” has been inserted after Eqs. (A5).

**Summary:**

**This work has significant of potential to present a novel methodology to combine simulations across multiple scales for biological applications but unfortunately the paper neither articulates the biological problem the authors are addressing clearly nor does it provide enough context for the novelty of the method. The mathematical and modeling work seems to be of great quality but the presentation needs to be improved.**

**Response:**

Thank you very much for your summary of the review results. One of the significant innovation in our approach is the introduction of a novel modeling component that includes cell division dynamics, including a sophisticated algorithm for cell division based on real-time changes in the intracellular signaling pathways, particularly the Hippo-YAP/TAZ pathway influencing on cell proliferation.
