# Supplementary material for: Molecular dynamics simulations of a multicellular model with cell-cell interactions and Hippo signaling pathway
Source: PLoS Comput Biol. 2024 Nov 11;20(11):e1012536. doi: 10.1371/journal.pcbi.1012536 (PMC11554158; doi:10.1371/journal.pcbi.1012536)
Supplement: Attachment — Submitted filename: ReplyLetter_PLOSCB20240906_HM_TU.docx [file pcbi.1012536.s011.docx]

**To: David van der Spoel, Academic Editor
Daniel Beard, Section Editor
PLOS Computational Biology**

**Subject: Response to Reviewers’ Comments for Manuscript [Manuscript ID:** **PCOMPBIOL-D-23-02102R1]**

**Dear Dr. van der Spoel and Dr. Beard,**

We would like to thank you and the reviewers for your thorough evaluation of our manuscript titled "Molecular dynamics simulations of a multicellular model with cell-cell interactions and Hippo signaling pathway." We have carefully considered the feedback provided and have made significant revisions to address the reviewers' comments. Below, we detail our responses and the changes made in the manuscript.

**Reviewer #1:**

1. **Comment:** *The main innovation of this work is the combination of classical mechanics to describe cellular motions, along with mass-action kinetics to describe intracellular signaling. The methodology seems novel. That said, the work appears incremental with respect to biology.*

**Response:** We appreciate the reviewer’s recognition of the novelty of our methodology. At the same time, we also understand the concern regarding its biological significance. To address this, we have added to Abstract: 'However, it is … proliferation.' Regarding the advancement, we included in Abstract: 'Finally, this mathematical model, … drug dosages.' Details of these points revealed by our 3D simulations have been added to Conclusion section by red words.

2. **Comment:** *I think it would be useful if the authors placed the intracellular dynamics and the inter-cellular dynamics in the context of execution dynamics (e.g.* [*https://www.nature.com/articles/s41540-019-0100-9*](https://www.nature.com/articles/s41540-019-0100-9) *or* [*https://journals.plos.org/ploscompbiol/article?id=10.1371/journal.pcbi.1004193*](https://journals.plos.org/ploscompbiol/article?id=10.1371/journal.pcbi.1004193)*) as I think this might be relevant to readers (at the very least mention in the discussion).*

**Response:** We agree with the reviewer that clearly distinguishing between cell dynamics and intracellular dynamics is crucial for understanding their relevance. To clarify these distinctions, we have improved Figure 1 and added the text in red to Materials and Methods and Discussion sections.

3. **Comment:** *Figure 1 is also very confusing. It would be useful if there was a schematic that depicts the overall methodology and then a workflow diagram, similar to that in figure 1 but simplified for readers.*

**Response:** We appreciate the feedback regarding Figure 1. In response, we have simplified Figure 1 to provide a clearer and more concise representation of our methodology. The revised figure now includes a more straightforward schematic of the workflow, highlighting the key steps in our approach while omitting nonessential details.

4. **Comment:** *Have the authors made all data and (if applicable) computational code underlying the findings in their manuscript fully available?*

**Response:**

We recognize the importance of transparency and reproducibility in scientific research. In response to the reviewer's comment, we have made the computational code freely accessible by providing a URL in the abstract. Additionally, we have included Appendix section in the revised manuscript that provides a comprehensive description of the computational model, detailing the key parameters, initial conditions, and numerical algorithms used. Appendix section also explains the methods for calculating cell interactions, growth, division, and reaction kinetics, offering an in-depth understanding of the computational processes involved in our study. By making both the code and supplementary information available, we aim to ensure that our methodology is clear and reproducible. Furthermore, all relevant data supporting our findings are provided in Table 1 and supplementary materials, ensuring transparency and verifiability of our work.

5. **Comment:** *Do you want your identity to be public for this peer review?*

**Response:** The reviewer has chosen to remain anonymous, and we respect their decision.

We have also carefully followed the additional instructions provided in the editorial comments, including ensuring that all figures meet PLOS requirements and confirming that all data and code are fully available as required.

Please find attached the revised manuscript, along with a tracked changes version for your reference. We hope that these revisions satisfactorily address the reviewers’ concerns. We believe that the manuscript has been significantly improved and that it is now ready for further evaluation.

Thank you once again for your time and consideration. We look forward to your feedback.

**Sincerely,**

**Toshihito Umegaki**

**Osaka University**

[**umegaki@sigmath.es.osaka-u.ac.jp**](mailto:umegaki@sigmath.es.osaka-u.ac.jp)
